# Multidimensional assessment of acne vulgaris: links between psychiatric symptoms, disordered eating, and dermatologic severity

**DOI:** 10.3389/fmed.2026.1790521

**Published:** 2026-04-17

**Authors:** Aydan Yazıcı, Ayşe Döndü

**Affiliations:** 1 Department of Dermatology, Faculty of Medicine, Aydın Adnan Menderes University, Aydın, Turkiye; 2 Department of Psychiatry, Faculty of Medicine, Aydın Adnan Menderes University, Aydın, Turkiye

**Keywords:** acne vulgaris, dermatologic severity, disordered eating, psychiatric symptoms, quality of life

## Abstract

**Introduction:**

Acne vulgaris is a common chronic inflammatory skin disease that extends beyond dermatologic symptoms to include psychosocial and behavioral consequences. Although the psychological impact of acne has been acknowledged, the interplay between psychiatric symptoms, eating behaviors, and quality of life remains underexplored.

**Objectives:**

To evaluate the associations between psychiatric symptoms, eating behavior patterns, and quality of life among patients with acne vulgaris compared to healthy controls.

**Methods:**

This case-control study included 120 patients with acne vulgaris and 100 sex-matched controls, age-adjusted in the analyses. Dermatologic assessment included the Global Acne Grading System (GAGS), acne scar characteristics, and the Turkish Acne Quality of Life (TAQoL) questionnaire. Psychiatric and behavioral evaluations included the Brief Psychiatric Rating Scale (BPRS), the Eating Attitudes Test (EAT), the Beck Depression and Anxiety Inventories, Night Eating Syndrome parameters, and the Eating Disorder Examination Questionnaire Turkish Short Form-13 item (EDE-Q-13). Statistical analyses were performed using SPSS 25.0, applying Mann-Whitney U, Pearson chi-square, logistic regression, and partial correlation tests with Benjamini-Hochberg correction. Significance was set at *p* < 0.05.

**Results:**

Patients with acne were predominantly female (80.0%) and significantly younger than controls (median age, 20 vs. 24 years, *p* < 0.001). A family history of acne was the strongest correlate of acne case status, a (OR = 5.5, 95% CI: 2.4–13.1, *p* < 0.001), with several familial cases increasing the risk up to 23-fold. Compared with controls, acne patients had significantly higher scores for psychiatric symptoms (BPRS, *p* < 0.001), depressive (*p* < 0.001), and anxiety symptoms (*p* < 0.001), and disordered eating (EAT, *p* = 0.001). Logistic regression identified binge-eating behavior (as measured by the EDE-Q-13 subscale) as an independent correlate of acne case status, with a higher odds ratio for acne cases (OR = 2.4, *p* < 0.001). Partial correlation analyses revealed that depression (*r* = 0.25, *p* = 0.008), anxiety (*r* = 0.29, *p* = 0.002), binge eating (*r* = 0.23, *p* = 0.013), and body dissatisfaction (*r* = 0.25, *p* = 0.009) were all positively correlated with poorer acne quality of life—night eating duration associated with the extent of trunk involvement (*r* = 0.20, *p* = 0.038).

**Conclusion:**

Acne vulgaris was associated with psychiatric distress, disordered eating, and impaired quality of life in this case-control sample. These findings support a broader psychosocial perspective, but causal inferences cannot be drawn from this cross-sectional design.

## Introduction

Acne vulgaris is among the most pervasive chronic inflammatory skin conditions, impacting a vast proportion of adolescents and many adults worldwide. Although often dismissed as a benign dermatologic nuisance, acne exacts a substantial psychosocial and behavioral toll that transcends the visible lesions (e.g., stigma, self-esteem issues, social withdrawal). Studies suggest strong links between acne and psychiatric comorbidities: a meta-analysis of 42 studies found higher rates of depression and anxiety in acne patients than controls (1–4). A large multicenter review estimated global prevalence of depression, anxiety, and suicidal ideation in acne patients at approximately 22%, 29, and 12%, respectively (5).

Cortisol release: The brain-skin axis has been proposed as a possible explanatory framework in prior literature; however, mechanistic links remain difficult to establish in observational studies (3, 6–8). Psychological distress may exacerbate sebum production and local cytokine milieu, further fueling a vicious cycle (8). In fact, a recent cross-sectional study from Italy reported that anxiety and depressive symptoms strongly correlated with acne exacerbation, particularly in individuals with lower resilience scores (9). Still, the relationship between acne severity and psychiatric burden is not always linear-for instance, one study found only a weak or absent association between objective lesion counts and depression or anxiety levels (3, 10).

Research is increasingly focusing on eating behaviors in relation to dermatology and psychology, beyond just mental health. While the role of diet in acne pathogenesis has been debated, accumulating evidence suggests that high-glycemic-load diets, dairy consumption, and other nutritional factors contribute to increased acne risk and severity (11–13). For example, meta-analyses have shown modest associations between skim milk intake and acne prevalence (12). Moreover, a 2025 study showed that adult acne patients had roughly 2.4 times higher odds of having a clinical eating disorder diagnosis (after adjusting for mood disorders or body image issues)-hinting at a behavioral or psychopathological pathway linking skin disease to disordered eating (14). However, studies using validated eating psychopathology instruments (e.g., EDE-Q, EAT, night-eating questionnaires) in acne populations remain scarce.

The quality of life (QoL) in individuals with acne has long been recognized as suffering. Acne can provoke appearance dissatisfaction, lower self-esteem, emotional distress, and impaired social functioning (e.g., avoidance, embarrassment) (3, 15–17). Some studies further show that psychological symptoms mediate the effect of acne on QoL, meaning that psychosocial distress may amplify how severely patients feel impacted (3, 18). The stigma dimension also plays a role: perceived or experienced stigmatization in acne patients is linked to higher psychological burden and lower life satisfaction (17).

Despite the growing interest, important gaps remain. Many prior works are cross-sectional, limiting causal inference; few integrate dermatologic severity, psychiatric symptoms, disordered eating, and QoL within a single model. The directionality (i.e., whether psychiatric distress leads to worse acne, or acne facilitates psychopathology) is rarely tested, and confounding by lifestyle factors (e.g., diet, stress, sleep) is not always controlled. A recent Mendelian randomization study failed to identify any evidence supporting a causal genetic relationship between acne and depression, anxiety, or other mental disorders. These findings suggest that the observed associations may be attributable to modifiable factors rather than to direct genetic effects on metabolism (19).

To address these gaps, we conducted a case-control study using a multidimensional framework that evaluated patients with acne vulgaris and matched controls across dermatologic variables (including GAGS scores and lesion distribution), psychiatric symptom burden (depression, anxiety, and general psychopathology), standardized eating-behavior measures (EAT, EDE-Q-13, and night-eating parameters), and acne-specific quality of life. The contribution of this study lies not in re-establishing the already recognized association between acne and psychological distress, but in integrating validated measures of eating behavior with psychiatric symptoms, acne-specific quality of life, and dermatologic severity within a single analytical framework. The specific aims were to: (i) compare psychiatric symptom burden, disordered eating, and quality of life between acne patients and controls; (ii) identify independent correlates of acne case status after adjustment for relevant confounders; and (iii) examine partial correlations between dermatologic severity, psychological measures, disordered eating, and quality-of-life outcomes. We hypothesized that patients with acne would show greater psychiatric distress and disordered eating than controls, and that these variables would be more closely associated with impaired quality of life than with global objective severity alone.

## Materials and methods

### Study design and participants

This case-control study was conducted between 1 January 2025 and 30 June 2025, at the Department of Dermatology, Aydın Adnan Menderes University Hospital, following ethics committee approval (Protocol Number: 2024/222). All participants provided written informed consent. This study adhered to the ethical principles outlined in the Declaration of Helsinki (2013 revision) and relevant national and institutional research regulations.

a total of 220 participants were included, comprising 120 patients with clinically diagnosed acne vulgaris and 100 healthy controls frequency-matched by sex, without any current or past dermatologic disease. A board-certified dermatologist established the diagnosis and grading of acne according to the Global Acne Grading System (GAGS). Exclusion criteria included systemic retinoid use within the past 6 months, pregnancy, major psychiatric disorders (e.g., psychosis, bipolar disorder, endocrine disorders, and use of medications known to affect mood or eating behavior (e.g., antidepressants, corticosteroids, appetite suppressants).

### Sociodemographic and clinical data

Demographic variables [age, sex, body mass index (BMI), educational level, smoking, alcohol consumption, and family history of acne] were collected through structured interviews and the institutional patient database. Body mass index (BMI) was calculated as weight (in kg) divided by height squared (in m^2^). Consistent with WHO global guidance, weight status categories were defined as underweight (<18.5 kg/m^2^), normal (18.5–24.9 kg/m^2^), overweight (25.0–29.9 kg/m^2^), and obesity (≥30.0 kg/m^2^) (20). Given the higher cardiometabolic risk at lower BMI in Asian populations, a sensitivity classification was performed using WHO Expert Consultation action points (≥23.0 for increased risk; ≥27.5 for high risk), without altering the primary WHO categorization (21).

Controls were frequency-matched by sex, while age was controlled for analytically by inclusion as a covariate in all adjusted models.

Clinical characteristics of acne were documented, including age of onset, disease duration, anatomic distribution (face, trunk), number of affected regions, and scarring patterns (ice-pick, boxcar, rolling, atrophic, keloidal). A total score is obtained by multiplying the coefficient of the most severe lesion in six anatomical regions on the face and trunk by the coefficient reflecting the pilosebaceous unit density. Accordingly, the acne score was evaluated as follows: 1–18, mild; 19–30, moderate; 31–38, severe; and 39 or higher, very severe (22). Acne scar severity was assessed using the Global Acne Scarring Grading System (GASGS), a qualitative assessment system based on scar morphology and the degree to which it can be camouflaged with makeup, and is graded from 1 to 4 (23).

### Psychiatric and behavioral assessments

All participants completed a standardized battery of validated self-report and clinician-rated instruments assessing psychiatric symptoms, eating behavior, and quality of life. Each scale was administered in its validated Turkish version by trained researchers in a quiet outpatient setting.

#### Brief Psychiatric Rating Scale (BPRS)

The BPRS was used to assess the overall level of psychopathology and psychological distress. It consists of 18 items evaluating domains such as anxiety, depression, hostility, somatic concern, and unusual thought content. Each item is rated on a seven-point response format scored from 0 to 6, yielding a total score range of 0–108. Higher scores reflect more severe psychiatric symptoms. The BPRS has demonstrated strong internal consistency (Cronbach’s α ≈ 0.81–0.90) and validity in Turkish clinical populations (24, 25).

#### Eating Attitudes Test (EAT-40)

The EAT-40 was employed to assess disordered eating attitudes and behaviors related to dieting, food preoccupation, and oral control. It comprises 40 items, each rated on a six-point scale from always (3) to never (0). Total scores range from 0 to 120, with scores of 30 or higher generally indicating a risk of clinically significant eating pathology. The EAT-40 provides subscale insights into dieting, bulimia, food preoccupation, and oral control (26). The Turkish version has been validated in clinical and non-clinical samples, demonstrating strong internal consistency (Cronbach’s α ≈ 0.88) and robust criterion validity against structured clinical interviews (27).

#### Beck Depression Inventory (BDI)

the BDI is a 21-item self-report questionnaire measuring the cognitive, affective, and somatic symptoms of depression. Each item is rated from 0 to 3, yielding a total score of 0 to 63. Conventional cut-off scores are 0–9 = minimal, 10–16 = mild, 17–29 = moderate, and 30–63 = severe depression (28). The Turkish version has been validated and widely used in dermatology-psychology research, with Cronbach’s α ≈ 0.88 (29).

#### Beck Anxiety Inventory (BAI)

The BAI is also a 21-item scale assessing somatic and subjective anxiety symptoms. Each item is rated on a four-point scale (0–3), with total scores ranging from 0 to 63. Severity is classified as minimal (0–7), mild (8–15), and severe (26–63) (30). The instrument exhibits excellent psychometric reliability (Cronbach’s α ≈ 0.92) in both clinical and non-clinical Turkish samples (31).

#### Night Eating Questionnaire (NEQ)

The NEQ assesses symptoms of Night Eating Syndrome (NES), including nocturnal awakenings, eating hyperphagia, mood-related eating, and morning anorexia. The 14 items are scored on a five-point Likert scale (0–4), with a total score ≥ 25 indicating clinically relevant NES. Subscales for evening hyperphagia and mood-related eating are also analyzed (32, 33).

#### Eating Disorder Examination Questionnaire (EDE-Q)

Eating Disorder Examination Questionnaire is a 28-item self-report instrument assessing core features of eating disorders over the past 28 days. It provides a global score and four subscales: (i) restraint, (ii) eating concern, (iii) shape concern, and (iv) weight concern. Items are rated from 0 (no days) to 6 (every day) (34). The Turkish adaptation, which is used in this study, is a 13-item short form which yields five subscales: (i) restraint (items1–3), (ii) shape/weight concern (items 4–5), (iii) body dissatisfaction (items 6–7), (iv) binge eating (items 8–10), (v) purging (items 11–13). Items were rated from 0 to 6 based on the number of days in the past 28 days. Each subscale score was calculated as the mean of its items, and a global score was derived according to the Turkish short-form specification, with high internal consistency (α > 0.90) (35).

To maintain transparency for international readers, please note that this five-subscale configuration differs from the original four-subscale structure of the 28-item EDE-Q.

#### Turkish Acne Quality of Life (TAQoL)

The acne-specific quality of life was assessed using the validated Turkish Acne Quality of Life (11 items; total score 0–44), where higher scores indicate worse quality of life, administered and scored according to the original manual (36). The Turkish version has demonstrated good reliability and construct validity in clinical samples. Note for compatibility: TAQoL is acne-specific and does not use DLQI impact bands; scores are interpreted as a continuous measure of burden.

#### Visual Analogue Scale (VAS)

Visual Analogue Scale was employed to capture each participant’s subjective perception of acne severity. Participants marked their perceived severity on a 10 cm horizontal line, anchored by 0 = no acne and 10 = worst imaginable acne (37).

### Statistical analysis

Statistical analyses were performed using IBM SPSS Statistics, Version 25.0 (IBM Corp., Armonk, NY, United States). With the available sample size (acne, *n* = 120; control, *n* = 100), the study had approximately 80% power to detect an odds ratio of about 2.0 at α = 0.05. Continuous variables are presented as mean ± standard deviation (SD), median (Q1–Q3), and range, whereas categorical variables are presented as counts and percentages. Normality was assessed using the Kolmogorov-Smirnov test. Categorical variables were compared using the Pearson chi-square test or Fisher-Freeman-Halton test with Monte Carlo simulation, as appropriate. For continuous variables, the independent-samples *t*-test was used for normally distributed data. In contrast, the Mann-Whitney U test with Monte Carlo simulation was used for non-normally distributed data.

Multiple testing was controlled using the Benjamini-Hochberg false discovery rate (*q* = 0.05). Given the number of psychological and behavioral variables examined, findings beyond the main variables of interest should be interpreted as exploratory despite Benjamini-Hochberg correction. For correlation families (e.g., TAQoL vs. psychiatric and eating-behavior indices), both raw and Benjamini-Hochberg-adjusted *p*-values were considered, and significance was defined at *q* = 0.05.

Binary logistic regression models were used to identify independent correlates of acne case status (acne vs. control), not to infer temporal or causal relationships. Results are presented as odds ratios (ORs) with 95% confidence intervals (CIs) per one-point increase in each scale score. Two adjusted models were constructed: Model 1 adjusted for age, sex, and BMI, and Model 2 additionally adjusted for educational level, smoking status, and alcohol consumption. Thus, age was included as a covariate in all adjusted logistic regression models. Collinearity was checked (all variance inflation factors were <3), linearity in the logit was assessed, and overall model fit was considered acceptable. Analyses were performed using a complete-case approach.

Partial correlation analyses were performed to explore associations among acne severity, quality of life, psychiatric symptoms, and eating-behavior variables while adjusting for confounders in two models. Age was included as a covariate in all adjusted partial correlation analyses. Correlation coefficients (r) and associated *p*-values are reported. All tests were two-sided, with α = 0.05, and exact *p*-values are provided where applicable (values < 0.001 are shown as “<0.001”).

### Ethical considerations

All procedures involving human participants were approved by the Adnan Menderes University Faculty of Medicine Non-Interventional Clinical Research Ethics Committee, and informed written consent was obtained from all subjects. Confidentiality of personal and medical data was maintained throughout the study. No financial incentives were provided.

## Results

### Clinical and demographic characteristics

The mean age at acne onset was 16.8 ± 4.0 years (median = 16 [14.5–18]), indicating that acne vulgaris predominantly begins during adolescence. The mean disease duration was 58.1 ± 48.8 months (median 48 [24–84]), suggesting chronicity in a large proportion of patients.

Facial involvement included an average of 3.4 ± 1.4 affected regions, whereas truncal involvement averaged 1.5 ± 1.2 areas. The mean GAGS score was 23.3 ± 7.5, with 48.3% of patients classified as moderate and 22.5% as severe. GASGS was primarily used to characterize the severity of scarring in the acne cohort.

Atrophic scars predominated, particularly ice-pick (80.8%) and boxcar (53.3%) scars; rolling scars were present in 21.7% of cases, whereas keloidal scars were uncommon (4.2%). TAQoL scores indicated marked acne-specific impairment in patients, with a mean of 14.6 ± 5.5 (median = 13 [IQR 11–18]). Subjective acne severity on the VAS was also higher, at 6.5 ± 1.9 (median 7 [IQR 5–8]), indicating that patients perceived a moderate-to-high burden (Table 1).

**TABLE 1:** Clinical features, GAGS scores, acne scar types, acne-related quality of life, and VAS values in acne patients.

	Mean (SD)	Median (Q1–Q3)
Age at acne onset (years)	16.8 (4.0)	16 (14.5–18)
Acne duration (months)	58.1 (48.8)	48 (24–84)
Number of facial acne localizations	3.4 (1.4)	4 (2–4)
Number of truncal acne localizations	1.5 (1.2)	2 (0–2)
GAGS (Global Acne Grading System)		
Forehead	4.2 (2.1)	4 (2–6)
Right cheek	5.4 (1.8)	6 (4–6)
Left cheek	5.3 (2.0)	6 (4–6)
Nose	1.0 (1.1)	1 (0–1)
Chin	2.3 (1.0)	3 (2–3)
Torso	5.2 (4.0)	6 (0–9)
Total score	23.3 (7.5)	22.5 (18–29)
	***n* (%)**	
GAGS total score		
No lesion (0)	0 (0.0)	
Mild (1–18)	34 (28.3)	
Moderate (19–30)	58 (48.3)	
Severe (31–38)	27 (22.5)	
Very severe (≥39)	1 (0.8)	
Scar – ice pick		
Absent	23 (19.2)	
Present	97 (80.8)	
Scar – boxcar		
Absent	56 (46.7)	
Present	64 (53.3)	
Scar – rolling		
Absent	94 (78.3)	
Present	26 (21.7)	
Scar – keloidal		
Absent	115 (95.8)	
Present	5 (4.2)	
Scar – atrophic		
Absent	104 (86.7)	
Present	16 (13.3)	
Degree of acne scar		
Macular	39 (32.5)	
Mild atrophic	52 (43.3)	
Moderate atrophic	26 (21.7)	
Severe atrophic	3 (2.5)	
	**Mean (SD)**	**Median (Q1–Q3)**
TAQoL score	14.6 (5.5)	13 (11–18)
VAS (Visual Analogue Scale)	6.5 (1.9)	7 (5–8)

GAGS, Global Acne Grading System; TAQoL, Turkish Acne Quality of Life (11 items; 0–44; higher = worse), TAQoL does not use DLQI banding; SD, standard deviation; Q1, 1st quartile; Q3, 3rd quartile.

### Sociodemographic features

The acne group was predominantly female (80.8%) and significantly younger than controls (median 20 vs. 24 years; *p* < 0.001). BMI did not differ significantly between groups (*p* = 0.698), although overweight status was more frequent among the controls (*p* = 0.019).

Educational level differed significantly (*p* < 0.001): high school graduates were more common in the acne group, whereas postgraduate education was more common in the control group. Smoking was lower in patients with acne (15.0% vs. 33%; *p* = 0.002), whereas alcohol consumption showed no difference.

A family history of acne was present in 43.3% of patients, compared with 14.0% of controls (*p* < 0.001). Severe familial acne was reported by 30.8% of patients, compared with 3.0% of controls (*p* < 0.001), respectively (Table 2).

**TABLE 2:** Sociodemographic characteristics and family history distributions of acne and control groups.

	Total (*n* = 220) *n* (%) or Med (Q1–Q3)	Control (*n* = 100) *n* (%) or Med (Q1–Q3)	Acne (*n* = 120) *n* (%) or Med (Q1–Q3)	*P*
Sex				**<0.001^c^**
Female	155 (70.5)	58 (58.0)	**97 (80.8)**	–
Male	65 (29.5)	**42 (42.0)**	23 (19.2)	–
Age (years)	22.0 (19.0–25.0)	**24.0 (20.5–29.0)**	20.0 (19.0–23.5)	**<0.001^u^**
BMI, kg/m^2^	22.3 (20.1–25.0)	22.4 (20.0–25.1)	21.9 (20.1–24.9)	0.698^u^
BMI				**0.049^c^**
Normal weight	139 (63.2)	59 (59.0)	80 (66.7)	ns
Overweight	48 (21.8)	**29 (29.0)**	19 (15.8)	0.019
Obese	33 (15.0)	12 (12.0)	21 (17.5)	ns
Educational level				**<0.001^ff^**
Illiterate	2 (0.9)	2 (2.0)	0 (0.0)	ns
Literate	2 (0.9)	2 (2.0)	0 (0.0)	ns
Primary education	5 (2.3)	2 (2.0)	3 (2.5)	ns
Secondary education	4 (1.8)	1 (1.0)	3 (2.5)	ns
High school education	45 (20.5)	13 (13.0)	**32 (26.7)**	0.012
Bachelor’s	140 (63.6)	62 (62.0)	78 (65.0)	ns
Master’s	15 (6.8)	**11 (11.0)**	4 (3.3)	0.025
Doctorate	7 (3.2)	**7 (7.0)**	0 (0.0)	<0.05
Smoking				**0.002 ^c^**
No	169 (76.8)	67 (67.0)	**102 (85.0)**	–
Yes	51 (23.2)	**33 (33.0)**	18 (15.0)	–
Alcohol consumption				0.356^c^
No	200 (90.9)	93 (93.0)	107 (89.2)	–
Yes	20 (9.1)	7 (7.0)	13 (10.8)	–
Familial acne history				<0.001^c^
No	154 (70.0)	**86 (86.0)**	68 (56.7)	–
Yes	66 (30.0)	14 (14.0)	**52 (43.3)**	–
Severity of family history				**<0.001^c^**
No	156 (70.9)	**86 (86.0)**	70 (58.3)	<0.001
Severe	40 (18.2)	3 (3.0)	**37 (30.8)**	<0.001
Not severe	24 (10.9)	11 (11.0)	13 (10.8)	ns

*^ff^*Fisher-Freeman-Halton (Monte Carlo).

*^c^*Pearson chi-square test (Monte Carlo); *Post-hoc* test; Benjamini-Hochberg correction.

*^u^*Mann-Whitney U test (Monte Carlo). Boldface indicates significant correlations (*p* < 0.05). Q1, 1st quartile; Q3, 3rd quartile, ns, not significant.

These findings suggest that genetic predisposition and demographic factors may be associated with the presence of acne vulgaris in this cohort.

### Psychiatric and behavioral assessments

Patients with acne had significantly higher BPRS scores (median = 18.0 [8.0–29.5]) than controls (8.0 [3.0–14.0]; *p* < 0.001).

Eating Attitudes Test scores were also higher (median = 13.0 vs. 10.0; *p* = 0.001), and 25.0% of acne patients exceeded the risk threshold, compared with 8.0% of controls.

Beck Depression Inventory scores were elevated in the acne group (median = 9.0 vs. 3.0; *p* < 0.001), with moderate to severe depression in 26.7% vs. 1.0%; *p* < 0.05.

BAI results followed a similar trend (median = 10.0 vs. 5.0; *p* < 0.001), with severe anxiety in 11.7% versus 1.0% (p = 0.002).

Night Eating Syndrome anxiety scores were higher in acne patients (median = 16.5 vs. 10.0; *p* < 0.001), and nocturnal eating behavior was more common (36.7% vs. 13.0%; *p* < 0.001). The duration of nocturnal eating behavior was also longer in the acne patients (0.5 vs. 0; *p* < 0.001).

On EDE-Q-13, acne patients scored significantly higher on dietary restraint, shape/weight concern, body dissatisfaction, and binge-eating subscales (all *p* < 0.005). The most significant differences were observed in shape/weight concern (*p* = 0.002) and binge eating (*p* < 0.001). The global EDE-Q-13 score was significantly higher in the acne group (median = 2.7 vs. 1.4; *p* = 0.001) (Tables 3, 4).

**TABLE 3:** Comparison of psychiatric evaluation, Eating Attitude Scale, Beck Depression and Anxiety Scales, Night Eating Syndrome, and EDE-Q-13 scores in acne and control groups.

	Total (*n* = 220) *n* (%) or med (Q1–Q3)	Control (*n* = 100) *n* (%) or med (Q1–Q3)	Acne (*n* = 120) *n* (%) or med (Q1–Q3)	*P*
Brief Psychiatric Rating Scale total	12.0 (5.5–23.0)	8.0 (3.0–14.0)	18.0 (8.0–29.5)	**<0.001^u^**
Severity distribution				**<0.001^ff^**
Minimal (≤31)	194 (88.2)	**100 (100.0)**	94 (78.3)	<0.05
Mild (32–53)	17 (7.7)	0 (0.0)	**17 (14.2)**	<0.05
Moderate (54–73)	7 (3.2)	0 (0.0)	**7 (5.8)**	<0.05
Severe (≥74)	2 (0.9)	0 (0.0)	2 (1.7)	ns
Eating Attitudes Test total	12.0 (8.0–17.0)	10.0 (8.0–14.0)	13.0 (9.0–19.5)	**0.001^u^**
Severity distribution				**0.001^c^**
Normal (≤19)	182 (82.7)	**92 (92.0)**	90 (75.0)	
Risky (≥20)	38 (17.3)	8 (8.0)	**30 (25.0)**	
Beck Depression Inventory total	6.5 (2.5–12.0)	3.0 (1.0–7.0)	9.0 (5.5–17.0)	
Severity distribution				**<0.001^ff^**
Minimal (0–9)	146 (66.4)	**85 (85.0)**	61 (50.8)	<0.001
Mild (10–16)	41 (18.6)	14 (14.0)	**27 (22.5)**	ns
Moderate (17–29)	27 (12.3)	1 (1.0)	**26 (21.7)**	<0.001
Severe (30–63)	6 (2.7)	0 (0.0)	**6 (5.0)**	<0.05
Beck Anxiety Inventory total	7.0 (2.0–14.5)	5.0 (1.0–8.0)	10.0 (4.0–17.0)	
Severity distribution				**<0.001^c^**
Minimal (0–7)	119 (54.1)	70 (70.0)	49 (40.8)	<0.001
Mild (8–15)	56 (25.5)	19 (19.0)	37 (30.8)	0.045
Moderate (16–25)	30 (13.6)	10 (10.0)	20 (16.7)	ns
Severe (26–63)	15 (6.8)	1 (1.0)	14 (11.7)	0.002
Night Eating Syndrome – anxiety	13.0 (9.0–18.5)	10.0 (7.5–13.0)	**16.5 (12.0–25.0)**	**<0.001^u^**
Night Eating Syndrome – mood-related eating (when sad)				**<0.001^ff^**
Early in the morning	28 (12.7)	20 (20.0)	8 (6.7)	0.003
Morning	7 (3.2)	3 (3.0)	4 (3.3)	ns
Afternoon	9 (4.1)	5 (5.0)	4 (3.3)	ns
Early evening	12 (5.5)	5 (5.0)	7 (5.8)	ns
Evening	14 (6.4)	5 (5.0)	9 (7.5)	ns
Night	57 (25.9)	13 (13.0)	44 (36.7)	<0.001
No change	93 (42.3)	49 (49.0)	44 (36.7)	ns
Duration of night eating (months)	0.0 (0.0–0.0)	0.0 (0.0–0.0)	**0.5 (0.0–1.0)**	**<0.001^u^**
EDE-Q-13 restraint	1.0 (0.0–2.7)	0.7 (0.0–2.0)	**1.0 (0.0–3.0)**	**0.014^u^**
EDE-Q-13 shape/weight concern	1.0 (0.0–3.0)	0.8 (0.0–1.5)	**1.5 (0.2–4.0)**	**0.002^u^**
EDE-Q-13 body dissatisfaction	1.0 (0.0–3.0)	1 (0.0–2.3)	**1.5 (0.0–4.0)**	**0.038^u^**
EDE-Q-13 binge eating	0.3 (0.0–1.0)	0.0 (0.0–0.7)	**0.3 (0.0–1.3)**	**<0.001^u^**
EDE-Q-13 purging	0.0 (0.0–0.0)	0.0 (0.0–0.0)	0.2 (0.0–0.5)	0.062^u^
EDE-Q-13 global score	2.2 (0.6–4.6)	1.4 (0.4–3.5)	**2.7 (0.8–5.9)**	**0.001^u^**

^ff^Fisher-Freeman-Halton (Monte Carlo).

^c^Pearson chi-square test (Monte Carlo); *Post-hoc* test: Benjamini-Hochberg correction.

^f^Fisher Exact test (exact).

^u^Mann-Whitney U test (Monte Carlo). Boldface indicates significant correlations (*p* < 0.05). EDE-Q-13, Eating Disorder Examination Questionnaire Turkish Short Form-13 item; Q1, 1st quartile; Q3, 3rd quartile; ns, not significant.

**TABLE 4:** Logistic regression analyses for variables distinguishing between acne and control groups: results of model 1 [adjusted for age, sex, and body mass index (BMI)] and model 2 (adjusted for age, sex, BMI, education level, smoking, and alcohol consumption).

Dependent variable: groups Reference group: acne	Model 1: adjusted for age, sex, BMI				Model 2: adjusted for age, sex, BMI, smoking, alcohol consumption, and educational level			
	*P*	Odds ratio	95% confidence interval for odds ratio		*P*	Odds ratio	95% confidence interval for odds ratio	
			Lower bound	Upper bound			Lower bound	Upper bound
Smoking (yes)	**0.008**	0.366	0.175	0.766	–	–	–	–
Alcohol consumption (yes)	0.559	1.373	0.474	3.974	–	–	–	–
Familial acne history (var)	**<0.001**	5.515	2.542	11.963	**<0.001**	5.541	2.351	13.059
Intensity of familial history								
No vs. severe	**<0.001**	16.255	4.483	58.936	**<0.001**	23.725	4.817	116.843
No vs. not severe	0.301	1.692	0.625	4.580	0.459	1.494	0.516	4.329
Brief Psychiatric Rating Scale (↑)	**<0.001**	1.081	1.047	1.117	**<0.001**	1.086	1.047	1.125
Eating Attitudes Test (↑)	**0.033**	1.048	1.004	1.094	**0.027**	1.056	1.006	1.108
Beck Depression Inventory (↑)	**<0.001**	1.145	1.081	1.212	**<0.001**	1.150	1.079	1.225
Beck Anxiety Inventory (↑)	**0.005**	1.059	1.018	1.102	**0.006**	1.065	1.018	1.113
Night Eating Syndrome – anxiety (↑)	**<0.001**	1.173	1.107	1.243	**<0.001**	1.182	1.109	1.260
EDE-Q-13 restraint (↑)	0.057	1.212	0.994	1.478	0.077	1.202	0.980	1.474
EDE-Q-13 shape/weight concern (↑)	0.108	1.150	0.970	1.363	0.182	1.131	0.944	1.356
EDE-Q-13 body dissatisfaction (↑)	0.449	1.065	0.905	1.253	0.751	1.029	0.863	1.226
EDE-Q-13 binge eating (↑)	**<0.001**	2.067	1.374	3.109	**<0.001**	2.384	1.524	3.729
EDE-Q-13 purging (↑)	0.157	1.701	0.815	3.553	0.233	1.596	0.741	3.438
EDE-Q-13 global score (↑)	**0.014**	1.179	1.034	1.344	**0.025**	1.171	1.020	1.345

Multivariable logistic regression (enter). Boldface indicates significant correlations (*p* < 0.05). EDE-Q-13, Eating Disorder Examination Questionnaire Turkish Short Form-13 item.

When considered collectively, these findings suggest that individuals with acne vulgaris not only experience dermatological challenges but also elevated levels of depression, anxiety, NES symptoms, and body image disturbances. The evidence underscores that acne vulgaris has significantly affected both quality of life and psychiatric wellbeing, highlighting the necessity for comprehensive, holistic patient assessment.

### Logistic regression analysis

Controlling first for age, sex, and BMI (Model 1) and then additionally for education, smoking, and alcohol consumption (Model 2), family history of acne emerged as the dominant correlate of case status: any family history increased the odds of being in the acne group by ≈ 5.5-fold (*p* < 0.001), and severe family history raised the odds by∼ 16–24-fold (*p* < 0.001). Among psychiatric and behavioral variables, BPRS, EAT-40, BDI, BAI, and NES-anxiety were each independently and positively associated with acne across models (*p* < 0.05). Within the EDE-Q-13 framework, the binge eating subscale was the most powerful behavioral predictor (Model 1, OR ≈ 2.07; Model 2, OR ≈ 2.38; both *p* < 0.001), whereas other EDE-Q-13 subscales were not independently related; the global score showed a modest positive association OR ≈ 1.17; *p* ≈ 0.01–0.03). Current smoking was inversely associated with acne in Model 1 (OR ≈ 0.366; *p* = 0.008) but attenuated to non-significance after full adjustment in Model 2. Collectively, these results indicate that familial predisposition and selected psychobehavioral variables, particularly binge eating, were independently linked to acne case status in this cohort, while smoking likely reflects residual confounding rather than a reproducible protective effect. These adjusted analyses were performed to reduce confounding related to the younger age distribution in the acne group (Table 4).

### Partial correlation analyses

Partial correlation analyses (adjusted for age, sex, and lifestyle variables) revealed that TAQoL was correlated positively with BPRS (*r* ≈ 0.24, *p* = 0.009), EAT (*r* ≈ 0.20, *p* = 0.031), BDI (*r* ≈ 0.27, *p* = 0.003), BAI (*r* ≈ 0.28, *p* = 0.002), EDE-Q-13 body dissatisfaction (*r* ≈ 0.26, *p* = 0.005), and EDE-Q-13 binge eating (*r* ≈ 0.23, *p* = 0.013). Global objective severity (GAGS total) showed no material partial correlations with psychiatric or eating-behavior measures, whereas acne-specific quality of life and subjective severity were more closely aligned with psychological variables. Exploratory site/chronology analyses indicated a small inverse association between purging and right-cheek GAGS (*r* ≈ −0.19, *p* ≈ 0.040), a positive association between purging and acne duration (*r* ≈ 0.37, *p* < 0.001), and positive associations between the number of truncal localizations and both binge-eating (*r* ≈ 0.30, *p* = 0.001) and NES-anxiety (*r* ≈ −0.18, *p* = 0.048). Other partial correlations were small and non-significant.

Taken together, these patterns indicate that perceived disease burden aligns with more psychiatric distress and disordered eating than global lesion counts. Clinically, brief screening for distress and binge-eating can flag high-impact patients when GAGS appears modest (Table 5).

**TABLE 5:** Partial correlation analyses adjusted for age, sex, and body mass index (BMI): associations between acne severity (GAGS), acne clinical variables, quality of life, psychiatric scales, and eating behaviors.

GAGS (Global Acne Grading System)								TAQoL score	Age at acne onset (year)	Acne duration (months)	Number of acne localizations	
Forehead		Right cheek	Left cheek	Nose	Jawline	Trunk	Total score				Facial	Truncal
Brief Psychiatric Rating Scale												
*r*	−0.177	0.114	0.065	0.054	−0.012	−0.047	−0.029	0.241	−0.085	0.117	0.136	−0.113
*P*	0.056	0.220	0.487	0.565	0.894	0.618	0.758	0.009	0.360	0.210	0.144	0.226
Eating Attitudes Test												
*r*	−0.057	−0.167	−0.157	0.158	0.174	0.012	−0.043	0.200	0.095	−0.095	0.015	−0.083
*P*	0.541	0.072	0.091	0.088	0.060	0.899	0.644	0.031	0.308	0.308	0.876	0.375
Beck Depression Inventory												
*r*	−0.158	0.087	0.019	0.029	0.038	0.047	0.007	0.268	−0.096	0.113	0.081	−0.008
*P*	0.089	0.352	0.843	0.753	0.686	0.611	0.940	0.003	0.301	0.224	0.383	0.929
Beck Anxiety Inventory												
*r*	0.015	0.126	0.023	0.073	−0.016	0.115	0.109	0.284	−0.072	0.080	0.058	0.064
*P*	0.876	0.176	0.807	0.435	0.867	0.216	0.240	0.002	0.438	0.391	0.533	0.490
Night Eating Syndrome – anxiety												
*r*	−0.066	−0.003	0.000	0.035	0.060	−0.009	−0.001	0.158	−0.086	0.111	0.178	−0.183
*P*	0.481	0.974	0.997	0.707	0.517	0.921	0.995	0.089	0.357	0.235	0.055	0.048
Duration of night eating (months)												
*r*	−0.066	0.045	−0.021	0.005	0.046	−0.079	−0.044	0.006	−0.006	0.004	0.149	−0.169
*P*	0.477	0.630	0.826	0.961	0.623	0.395	0.641	0.946	0.947	0.969	0.109	0.068
EDE-Q-13 restraint												
*r*	0.056	0.009	−0.246	−0.021	−0.101	−0.008	−0.063	−0.033	−0.029	0.051	−0.082	0.108
*P*	0.550	0.926	**0.008**	0.821	0.277	0.929	0.499	0.726	0.760	0.586	0.379	0.246
EDE-Q-13 shape/weight concern												
*r*	−0.029	−0.040	−0.063	0.001	−0.005	0.029	−0.005	0.145	0.069	−0.057	0.005	0.002
*P*	0.759	0.670	0.502	0.993	0.960	0.756	0.955	0.120	0.457	0.543	0.962	0.982
EDE-Q-13 body dissatisfaction												
*r*	−0.065	0.007	−0.008	0.036	0.000	0.040	0.001	0.259	−0.088	0.090	0.055	0.019
*P*	0.485	0.937	0.935	0.698	0.997	0.665	0.990	**0.005**	0.344	0.333	0.555	0.841
EDE-Q-13 binge eating												
*r*	0.007	0.012	0.016	0.181	0.042	−0.030	0.027	0.229	0.029	−0.011	0.060	−0.299
*P*	0.942	0.901	0.867	0.051	0.651	0.745	0.773	**0.013**	0.756	0.908	0.517	**0.001**
EDE-Q-13 purging												
*r*	−0.074	−0.190	−0.170	0.368	0.001	0.001	−0.055	0.089	0.154	−0.144	−0.112	−0.058
*P*	0.428	**0.040**	0.067	**0.000**	0.992	0.991	0.556	0.337	0.098	0.122	0.230	0.536
EDE-Q-13 global score												
*r*	−0.022	−0.014	−0.084	0.059	0.014	0.062	0.024	0.156	−0.001	0.029	0.069	−0.032
*P*	0.811	0.878	0.368	0.527	0.885	0.505	0.798	0.093	0.994	0.758	0.459	0.736
VAS (Visual Analogue Scale)												
*r*	0.154	0.213	0.198	0.153	0.070	0.002	0.163	0.276	−0.051	0.003	0.172	0.052
*P*	0.097	**0.021**	**0.033**	0.099	0.452	0.983	0.080	**0.003**	0.585	0.973	0.063	0.576

Partial correlations are adjusted for sex, age (in years), and body mass index (BMI, in kg/m^2^). Both raw and Benjamini-Hochberg FDR-adjusted *p*-values are reported; *q* = 0.05, TAQoL, Turkish Acne Quality of Life (11 items; 0–44; higher = worse); EDE-Q-13, Eating Disorder Examination Questionnaire Turkish Short Form-13 item. Boldface indicates significant correlations (*p* < 0.05).

Additionally, in fully adjusted partial correlations (Table 6), the TAQoL-psychopathology pattern remained robust (BPRS/BDI/BAI and EDE-Q-13 body dissatisfaction and binge eating all positive). At the same time, objective severity stayed essentially null, except for VAS aligning with cheek GAGS (right *r* ≈ 0.28, left *r* ≈ 0.24) and a small inverse BPRS-forehead link (*r* ≈ −0.19). These signals indicate robustness to lifestyle/familial confounding (Benjamini-Hochberg FDR maintained at *q* = 0.05). Clinically, TAQoL tracked patient-perceived burden more closely than GAGS, supporting the routine use of psychosocial screening alongside dermatologic assessment.

**TABLE 6:** Partial correlation analyses adjusted for age, sex, body mass index (BMI), education level, smoking and alcohol consumption, and family history of acne: associations between acne severity (GAGS), acne clinical variables, quality of life, psychiatric scales, and eating behaviors.

GAGS (Global Acne Grading System)								TAQoL score	Age at acne onset (years)	Acne duration (months)	Number of acne localizations	
Forehead		Right cheek	Left cheek	Nose	Jawline	Trunk	Total score				Facial	Truncal
Brief Psychiatric Rating Scale												
*r*	−0.188	0.116	0.076	0.054	−0.011	−0.057	−0.039	0.250	−0.078	0.106	0.117	−0.134
*P*	0.046	0.221	0.422	0.569	0.906	0.551	0.681	0.008	0.412	0.265	0.215	0.158
Eating Attitudes Test												
*r*	−0.056	−0.169	−0.157	0.175	0.172	−0.002	−0.055	0.181	0.119	−0.122	−0.021	−0.088
*P*	0.558	0.074	0.096	0.064	0.069	0.983	0.560	0.055	0.210	0.199	0.829	0.354
Beck Depression Inventory												
*r*	−0.162	0.106	0.024	0.037	0.032	0.037	0.000	0.247	−0.073	0.090	0.050	−0.006
*P*	0.087	0.263	0.798	0.696	0.734	0.701	0.996	0.008	0.442	0.345	0.598	0.950
Beck Anxiety Inventory												
*r*	0.022	0.118	0.023	0.090	−0.026	0.104	0.098	0.285	−0.070	0.080	0.033	0.055
*P*	0.814	0.212	0.812	0.341	0.785	0.272	0.303	0.002	0.460	0.397	0.730	0.560
Night Eating Syndrome – anxiety												
r	−0.050	−0.002	−0.007	0.068	0.043	−0.029	−0.015	0.120	−0.069	0.104	0.148	−0.180
*P*	0.600	0.986	0.937	0.472	0.649	0.757	0.873	0.204	0.468	0.271	0.118	0.057
Duration of night eating (months)												
*r*	−0.061	0.052	−0.002	0.012	0.095	−0.050	−0.013	0.042	0.001	−0.007	0.195	−0.173
*P*	0.523	0.585	0.984	0.900	0.315	0.597	0.892	0.660	0.995	0.940	0.038	0.067
EDE-Q-13 restraint												
*r*	0.066	0.036	−0.235	−0.011	−0.071	0.013	−0.037	−0.038	0.001	0.020	−0.077	0.128
*P*	0.489	0.704	0.012	0.908	0.452	0.895	0.696	0.687	0.994	0.833	0.417	0.178
EDE-Q-13 shape/weight concern												
*r*	−0.052	−0.010	−0.049	−0.031	0.017	0.048	0.015	0.169	0.085	-0.084	0.025	0.006
*P*	0.584	0.914	0.609	0.744	0.854	0.614	0.873	0.074	0.373	0.375	0.795	0.950
EDE-Q-13 body dissatisfaction												
*r*	−0.080	0.044	−0.001	0.025	−0.001	0.040	0.007	0.246	−0.066	0.063	0.043	0.032
*P*	0.402	0.647	0.994	0.796	0.989	0.676	0.945	0.009	0.489	0.506	0.650	0.734
EDE-Q-13 binge eating												
r	0.019	0.005	0.015	0.204	0.044	−0.033	0.026	0.233	0.033	−0.011	0.057	−0.302
*P*	0.843	0.959	0.877	0.031	0.641	0.732	0.783	0.013	0.726	0.910	0.548	0.001
EDE-Q-13 purging												
*r*	−0.118	−0.177	−0.151	0.341	0.011	−0.004	−0.062	0.100	0.185	−0.201	−0.148	−0.090
*P*	0.212	0.061	0.110	0.000	0.905	0.969	0.512	0.293	0.050	0.033	0.118	0.344
EDE-Q-13 global score												
*r*	−0.037	0.018	−0.075	0.045	0.019	0.065	0.032	0.146	0.024	−0.002	0.061	−0.025
*P*	0.694	0.852	0.430	0.638	0.846	0.494	0.740	0.123	0.804	0.982	0.520	0.794
VAS (Visual Analogue Scale)												
*r*	0.125	0.282	0.239	0.124	0.081	−0.009	0.169	0.262	0.000	−0.076	0.132	0.042
*P*	0.187	0.002	0.011	0.191	0.391	0.925	0.073	0.005	0.997	0.425	0.163	0.658

Partial correlations are adjusted for sex, age (in years), body mass index (BMI, in kg/m^2^), educational level, smoking, alcohol consumption, and family history of acne. Both raw and Benjamini-Hochberg FDR-adjusted *p*-values are reported. EDE-Q-13: Eating Disorder Examination Questionnaire Turkish Short Form-13 item; TAQoL, Turkish Acne Quality of Life (11 items; 0–44; higher = worse). Boldface indicates significant correlations (*p* < 0.05).

## Discussion

Building upon the multidimensional findings of this study, acne vulgaris extends well beyond cutaneous manifestations, encompassing interrelated psychiatric, behavioral, and hereditary domains. The present results showed that patients with acne had higher levels of depression, anxiety, general psychopathology, and disordered eating behaviors, and that these variables were associated with acne status and perceived disease burden. Because this was a cross-sectional design, these findings should be interpreted as associations rather than causal or pathophysiologic relationships. These associations underscore that acne’s impact extends beyond visible lesions to encompass broader biopsychosocial processes influencing mental health and daily functioning. As shown in multivariate analyses, family history remained a dominant signal for case status, while binge-eating (EDE-Q-13 Binge Eating) emerged as the clearest behavioral correlate; smoking’s inverse association in the minimally adjusted model was not retained after full adjustment. These regression models should be interpreted as identifying correlates of acne case status within this sample rather than causal determinants of disease.

Elevated depression and anxiety scores align with broader literature documenting an association between acne and internalizing psychopathology (4). Although the brain-skin axis has been proposed in prior literature as a possible explanatory framework, our data do not directly test neuroendocrine or inflammatory mechanisms and therefore should be interpreted in associative rather than mechanistic terms (37–39). Although some studies report weak or absent relationships between objective measures of acne severity and psychological distress, our partial correlations likewise showed no material association between global objective severity (GAGS total) and psychopathology/QoL. In contrast, subjective severity (VAS) was more closely related to selected site-specific measures, consistent with prior reports that objective lesion counts do not always parallel psychological burden (10). The observed correlations were modest in magnitude but directionally consistent, suggesting that perceived burden may align more closely with psychological distress than global lesion counts do. In this study, GASGS served primarily as a descriptive indicator of scarring rather than a central psychodermatologic variable. The observed correlations between psychiatric symptom scores and acne-specific quality of life (TAQoL) were modest in magnitude (*r* ≈ 0.25–0.29) but directionally consistent, suggesting that psychological distress was associated with patients’ perception of disease burden. These modest but directionally consistent correlations suggest that psychological distress may align with patients’ perception of disease burden. Prior psychodermatology literature has discussed neuroendocrine and inflammatory pathways as possible background mechanisms, but such pathways were not measured in the present study and should not be inferred from these findings (4). Similarly, significant relationships between binge-eating, body dissatisfaction, and night-eating behaviors with both acne extent and truncal distribution raise exploratory questions regarding possible metabolic or circadian correlates discussed in the literature; however, such pathways were not measured in the present study and should not be inferred from our findings (40, 41). Within this study, binge eating showed the strongest association with case status and QoL, whereas purging behaved as a frequency marker tied to chronology/topography (positively with longer disease duration and modestly/inversely with right cheek GAGS) rather than to global severity. Although false discovery rate correction was applied, some site-specific and exploratory correlations should be interpreted cautiously and require replication.

Disordered eating, particularly binge-eating behavior, emerged as a relevant correlate in our analyses and may represent an underexplored dimension of psychodermatologic burden in acne (14). Prior literature has proposed that high-glycemic-load diets, irregular eating patterns, and related metabolic pathways such as insulin/IGF-1 or mTORC1 signaling may contribute to acne biology (40, 42, 43). However, these pathways were not directly measured in the present study and therefore should not be inferred from our findings. Likewise, the observed association between night-eating duration and truncal involvement should be interpreted cautiously and viewed as hypothesis-generating rather than mechanistic evidence. Rather than demonstrating a causal or pathophysiologic role, our results suggest that psychiatric distress and maladaptive eating behaviors may coexist with acne and shape patient-reported burden in clinically relevant ways (38, 44). From a practical standpoint, brief screening for depression, anxiety, and disordered eating may help identify patients with high psychosocial burden, even when global lesion counts are not striking (2, 15).

Moreover, the observed link between night-eating behaviors and truncal acne distribution should be considered exploratory and hypothesis-generating. While prior literature has discussed circadian and metabolic pathways in relation to acne, no hormonal, metabolic, or inflammatory biomarkers were collected in this study; therefore, mechanistic interpretations are not warranted (40, 41, 45, 46). Collectively, these findings suggest that psychiatric distress and maladaptive eating behaviors are clinically relevant co-occurring features in patients with acne and may contribute to patient-reported burden (38, 44). Clinically, this supports coupling dermatologic care with the brief screening for depression/anxiety and eating pathology (e.g., EAT, EDE-Q-13 binge eating) even when GAGS appears modest.

The prominence of family history as the most potent correlate of acne status in our models, underscores the relevance of hereditary background in acne vulnerability. Several studies have confirmed familial clustering of acne and identified multiple genetic loci associated with susceptibility to acne, including variants in inflammatory cytokine genes and the androgen receptor (38, 47, 48). Conversely, the inverse association of smoking in the minimally adjusted model, which lost significance after full adjustment, aligns with meta-analytic work suggesting that the protective effect of smoking on acne is likely artifactual or confounded rather than causal (49). Taken together, the full-adjusted pattern suggests that hereditary load and psychobehavioral burden, rather than smoking, are the primary, reproducible correlates of acne status in this cohort.

From a clinical perspective, these findings do not by themselves justify a change in treatment algorithms, but they do suggest that psychosocial assessment may add value in selected patients with acne. In particular, brief screening for depression, anxiety, and disordered eating may help identify individuals with disproportionate patient-reported burden, especially when objective lesion counts and psychosocial distress appear discordant. A structured family history prompt at intake may also be useful for clinical characterization. These observations support consideration of multidisciplinary input when psychosocial burden is prominent, but interventional studies are needed to determine whether such approaches improve dermatologic or patient-reported outcomes (2, 15).

This study undoubtedly has some limitations that need to be acknowledged. Because this was a cross-sectional case-control study, temporal direction could not be established; psychiatric distress and disordered eating may precede acne, result from acne, or relate bidirectionally. Although validated self-report instruments were employed, no hormonal, metabolic, or inflammatory biomarkers were measured; therefore, mechanistic interpretations remain speculative (10). Despite age adjustment, residual confounding by age or life-stage factors cannot be excluded. Additionally, although the sample size was substantial and included matched controls, single-center recruitment may limit generalizability. Future work should test whether targeted reduction of distress and binge-eating can lower TAQoL and improve adherence/clinical outcomes within stepped, integrative care pathways.

Overall, our findings suggest that acne vulgaris is associated with hereditary background, psychiatric distress, disordered eating, and impaired acne-specific quality of life (4). A central observation of this study is the discordance between global objective severity and patient-reported psychosocial burden. Recognition of these interconnections underscores the need for an integrative model of care that jointly addresses dermatologic, psychiatric, and nutritional domains. Routine assessment of mental-health symptoms and eating behaviors may enable early identification of vulnerable individuals and optimize treatment adherence. To sum up, perceived burden aligns more closely with psychiatric distress and disordered eating than with global lesion counts, reinforcing the clinical value of psychosocial screening along with standard dermatologic management.

Beyond observational design, interventional trials targeting psychiatric distress and disordered eating, embedded within dermatologic care pathways, are warranted to determine whether modifying these domains yields measurable improvements in clinical severity and patient-reported outcomes.

Future research should prioritize randomized, interventional trials that target psychiatric distress and disordered eating (e.g., collaborative care, CBT-based, or dietitian-led programs) to test whether integrative management improves both dermatologic endpoints and quality of life. Such trials would also pre-specify site-specific outcomes (e.g., cheek/trunk indices) and TAQoL as co-primary endpoints to capture both topography and patient-reported burden.

## Data Availability

The raw data supporting the conclusions of this article will be made available by the authors, without undue reservation.
